# Intravenous Immunglobulin Binds Beta Amyloid and Modifies Its Aggregation, Neurotoxicity and Microglial Phagocytosis *In Vitro*

**DOI:** 10.1371/journal.pone.0063162

**Published:** 2013-05-16

**Authors:** Susann Cattepoel, Alexander Schaub, Miriam Ender, Annette Gaida, Alain Kropf, Ursula Guggisberg, Marc W. Nolte, Louis Fabri, Paul A. Adlard, David I. Finkelstein, Reinhard Bolli, Sylvia M. Miescher

**Affiliations:** 1 CSL Behring AG, Bern, Switzerland; 2 CSL Behring GmbH, Marburg, Germany; 3 CSL Limited, Melbourne, Australia; 4 Mental Health Research Institute, Parkville, Australia; Virginia Commonwealth University, United States of America

## Abstract

Intravenous Immunoglobulin (IVIG) has been proposed as a potential therapeutic for Alzheimer's disease (AD) and its efficacy is currently being tested in mild-to-moderate AD. Earlier studies reported the presence of anti-amyloid beta (Aβ) antibodies in IVIG. These observations led to clinical studies investigating the potential role of IVIG as a therapeutic agent in AD. Also, IVIG is known to mediate beneficial effects in chronic inflammatory and autoimmune conditions by interfering with various pathological processes. Therefore, we investigated the effects of IVIG and purified polyclonal Aβ -specific antibodies (pAbs-Aβ) on aggregation, toxicity and phagocytosis of Aβ *in vitro*, thus elucidating some of the potential mechanisms of action of IVIG in AD patients. We report that both IVIG and pAbs-Aβ specifically bound to Aβ and inhibited its aggregation in a dose-dependent manner as measured by Thioflavin T assay. Additionally, IVIG and the purified pAbs-Aβ inhibited Aβ-induced neurotoxicity in the SH-SY5Y human neuroblastoma cell line and prevented Aβ binding to rat primary cortical neurons. Interestingly, IVIG and pAbs-Aβ also increased the number of phagocytosing cells as well as the amount of phagocytosed fibrillar Aβ by BV-2 microglia. Phagocytosis of Aβ depended on receptor-mediated endocytosis and was accompanied by upregulation of CD11b expression. Importantly, we could also show that Privigen dose-dependently reversed Aβ-mediated LTP inhibition in mouse hippocampal slices. Therefore, our *in vitro* results suggest that IVIG may have an impact on different processes involved in AD pathogenesis, thereby promoting further understanding of the effects of IVIG observed in clinical studies.

## Introduction

Alzheimer's disease (AD) is the most common form of dementia in the aging population. It is characterized by progressive memory deficits and cognitive impairment. The formation and accumulation of the amyloid-beta (Aβ) peptide and its toxic aggregates is thought to be the initiating event that is followed by synaptic dysfunction, inflammation and eventually neuronal death [1], [2]. Therefore, numerous strategies to prevent Aβ aggregation and toxicity are currently studied for potential therapy of AD.

Immunotherapy with monoclonal anti-Aβ antibodies demonstrated activity in transgenic mouse models [3], [4] and a variety of anti-Aβ antibodies are currently explored as potential therapeutics for AD [5]. The Phase III clinical trials for the monoclonal antibodies Bapineuzumab (J&J/Pfizer) and Solanezumab (Eli Lilly) have recently been completed and for both it was reported that the primary endpoints, cognitive and functional, were not met. Also, a polyclonal intravenous immunoglobulin (IVIG) preparation (Gammagard liquid; Baxter) is still in clinical Phase III trials (ClinicalTrials.gov). Gammagard liquid and other IVIG products are commercial preparations of purified human IgG manufactured from pooled plasma from thousands of donors [6]. A Phase II study in a small number of AD patients showed maintenance of cognitive function in subjects treated with 0.4 g IVIG/kg/2weeks [7]. The extended Phase II revealed that those who were treated with 0.4 g IVIG/kg/2 weeks for the full 36 months had the best outcome, with no decline in standard cognitive tests (Relkin et al., Alzheimer's Dementia 2012; 8(4) Supplement, P589). A small prospective clinical trial of IVIG therapy in AD patients, showed that IVIG-treatment increased Aβ in serum and decreased Aβ in CSF, and maintained cognitive function as compared to saline-treated control AD patients [8]. Another phase II dose-finding trial of IVIG for treatment of mild-moderate AD found significantly increased plasma Aβ40 in the 0.4 g/kg every two weeks patient group compared to placebo group [9].

Natural, polyclonal anti-Aβ antibodies have been detected in various IVIG preparations [6], [10]. Therefore, one of the potential mechanisms of action proposed for IVIG in AD is the direct binding of Aβ by natural anti-Aβ antibodies. However, other possible mechanisms relate to the immunomodulatory and anti-inflammatory effects of IVIG [11]. Neuroinflammation characterized by activated microglia and upregulation of a variety of inflammatory mediators, such as cytokines, chemokines and reactive oxygen species (ROS), is a prominent feature of AD [12]. Thus, IVIG might also influence inflammatory processes in the brain including modulation of microglial activation and increase of microglial phagocytosis of fibrillar Aβ [13], [14]. Furthermore, we found that, apart from anti-Aβ antibodies, IVIG contains antibodies against other disease-relevant proteins, such as Tau, pTau, RAGE and PrPc (Schaub et al., Neurodegenerative Dis. 2011; 7(4) Supplement, Page S670). Therefore, in contrast to monoclonal antibodies that recognize only one specific linear or conformational epitope in Aβ and polyclonal anti-Aβ antibodies that recognize a variety of different linear and conformational epitopes in Aβ [15] IVIG recognize several different potentially disease relevant targets [16]. This suggests IVIG could be a promising therapeutic for a multifactorial disease such as AD.

In the current study we demonstrate that the IVIG preparation Privigen and the purified pAbs-Aβ both bound specifically to Aβ, inhibited Aβ-aggregation and Aβ-mediated toxicity. Both preparations prevented the binding of Aβ to primary cortical neurons and increased the phagocytosis of fibrillar Aβ by BV-2 cells, which correlated with increased expression of CD11b. In addition, Privigen completely reversed Aβ-mediated LTP inhibition in mouse hippocampal slices. Our results indicate that IVIG could have the potential to improve certain AD related pathologies by multiple mechanisms.

## Materials and Methods

### IVIG preparations

For all assays in this study the IVIG product Privigen® 10% (CSL Behring AG, Bern, Switzerland) was used. Privigen is a ready-to-use, sterile, 10% protein liquid preparation of polyvalent human immunoglobulin G (IgG) for intravenous administration [17], [18]. IVIG is derived from large pools of human plasma (10,000–60,000 of donations) and therefore represents the antibody spectrum present in the donor population [19].

The Privigen F(ab′)_2_ fragments were produced by pepsin digestion of IVIG (Privigen) in acetate buffer pH 4.0 for 2 hours at 37°C. The reaction was stopped by adding 2 M Tris base until a pH of 8 was reached.

Privigen Fc fragments were prepared from IVIG (Privigen) by papain digestion and purification by ion exchange and size exclusion chromatography. Remaining Fab fragments were eliminated by Fab-specific affinity chromatography (Athens Research Technologies, Athens, GA). Finally, Privigen Fc fragments were polished by EndoTrapHD (Hyglos, Regensburg, Germany) resulting in endotoxin levels below 5 pg/ml.

The purity of the fragments was confirmed by SDS-PAGE and endotoxin levels were determined by commercially available chromogenic LAL test (Lonza, Allendale, NJ). An endotoxin level of less than 1 ng/ml was previously tested and shown not to interfere with the assays used.

### Affinity chromatography

Natural antibodies specific for oligomeric Aβ were isolated from Privigen by affinity chromatography using a column containing UltraLink Biosupport resin (Thermo Fisher Scientific, Waltham, MA) coupled with cross-linked Aβ oligomers in coupling buffer (0.2 M sodium carbonate, 0.6 M sodium citrate, pH 10) for 3 h at RT and washed, blocked with 1 M monoethanolamine, pH 9. For affinity purification Privigen was diluted 1∶70 in PBS and loaded onto the column. The column was washed with PBS and polyclonal Aβ-specific antibodies (pAbs- Aβ) were eluted with 0.1 M glycine, 2% acetic acid, pH 2.2 and immediately neutralized to pH 7. The yield of pAbs- Aβ was in the range of 0.1% of total IVIG load. Each batch of the purified Aβ-specific polyclonal antibodies (pAbs-Aβ) was routinely tested in ELISA for binding capacity and endotoxin levels.

### Preparation and characterization of Aβ42

Recombinant Aβ42 peptide was obtained as ultra pure hexafluoro-2-propanol (HFIP) film from rPeptide (Bogart, GA) and reconstituted in HFIP, which was evaporated with a constant stream of nitrogen. The peptide was then again resuspended in HFIP and aliquoted in Protein LoBind tubes (Eppendorf, Hamburg, Germany). Again HFIP was evaporated with nitrogen, the tubes were snap frozen in liquid nitrogen and aliquots were stored at −70°C. For experimental use the HFIP film of the peptide was dissolved in 10 mM NaOH (pH 12), which keeps Aβ42 in its monomeric form. After neutralization with an equivalent amount of 10 mM HCl (pH 2) the aggregation process was started [20].

Aβ oligomers were prepared according to Lambert et al. [21] with some modifications. Briefly, Aβ was dissolved as described above, PBS added and incubated for 4 h at 30°C. The oligomers were stabilized by cross-linking with 25 mM peroxynitrite for 20 min at room temperature (RT) [22]. The oligomers were characterized by SDS-PAGE and Atomic Force Microscopy (AFM).

Fibrillar FITC-Aβ42 was prepared by incubating FITC-labelled Aβ42 (rPeptide) with monomeric Aβ42 (1∶8) in 1× PBS over night at 37°C and shaking at 300 rpm.

For SDS-PAGE equal amounts of protein were separated on a 4–12% Bis-Tris gel (Invitrogen, Carlsbad, CA) and silver stained with SilverQuest Silver Staining Kit (Invitrogen) according to the manufacturer's protocol. Signals were detected using the ImageQuant LAS4000 (GE Healthcare, Waukesha, WI).

For Atomic Force Microscopy (AFM) glass slides were incubated in 2% Hellmanex II (Hellma, Müllheim, Germany) at 50°C for 60 min, washed 15 min with ultra pure water, then incubated 60 min in a solution of 2.8 M NaOH and 53% ethanol (EtOH) [23]. Subsequently glass slides were washed 2 times 15 min in ultra pure water, sonicated 3 times 20 min in ultra pure water and dried with nitrogen. Samples of 10 µl were applied on slides, incubated for 10 min, washed with ultra pure water and again dried in a nitrogen flow. Probes were measured in tapping mode using a NSC15/AIBS cantilever (Mikromasch, Tallinn, Estonia) on a NanoWizard II (JPK, Berlin, Germany) and images were processed with the NanoWizard IP software.

### ELISA binding assay and competition ELISA

Binding of Privigen to Aβ42 oligomers was measured by direct ELISA in Nunc Maxisorp 96F plates (Nunc, Penfield, NY). The oligomers were coated at a concentration of 0.33 µM over night at 4°C and blocked with monoethanolamine (1%, pH 7.5). Privigen, Privigen Fc fragment, pAbs-Aβ and monoclonal anti- Aβ antibody 6E10 (Covance, Princeton; NJ) (positive control) were diluted in Low-Cross Buffer (Candor Biosciences GmbH, Wangen, Germany) and incubated for 2 h at RT. Detection antibodies anti-mouse (1∶2000, Dako, Glostrup, Denmark) and anti-human IgG HRP (1∶2000, Dako) diluted in Superblock (Thermo Fisher Scientific) 1∶5 PBS Tween 0.05% (SPBST) were incubated for 1 h at RT. The ELISA was developed with TMB ultra sensitive substrate (Fitzgerald Industries, Acton, MA) and the reaction was stopped with sulfuric acid (1M). The absorbance was measured at 450 nm using the multilabel reader EnVision Xcite (Perkin Elmer, Waltham, MA).

For the competition analysis the plate was coated with Aβ42 oligomers as previously described. Privigen (100 µg/ml), pAbs-Aβ (25 µg/ml), and molar equivalent of Privigen Fc fragment (33 µg/ml), Myeloma Mix (The Binding Site, Birmingham, UK) (100 µg/ml) and 6E10 (1 µg/ml) were pre-incubated with 20-fold excess of Aβ for 4 h at RT and shaking at 350 rpm. The pre-incubated mixtures were added to the coated ELISA plate for 1 h at RT. After incubation these mixtures were transferred to another Aβ42-coated plate and treated identically, in order to confirm that the binding equilibrium was not disturbed. Both plates were subsequently treated as described for the ELISA binding assay.

### BioLayer Interferometry

BioLayer Interferometry (BLI) is a label-free optical technology for measuring biomolecular interactions on a biosensor tip. The assays were measured in a 96-well format on an Octet QKe device (FortéBio Inc., Menlo Park, CA), where the biosensor tip was moved from well to well for incubation [24]. For the pre-equilibrium the Streptavidin-coated biosensor tip was incubated in PBS (pH 7.4) or 1× Kinetic Buffer (KB, FortéBio Inc.) for at least 600 s, the tip was then loaded with monomeric biotinylated-Aβ42 (rPeptide) (25 µg/ml in PBS, pH 7.4 or KB) for 30 s and quenched with Biocitin (FortéBio Inc.) for 300 s. After the generation of a 2^nd^ baseline in PBS (pH 7.4) or KB for 300 s, the association step with pAbs-Aβ or 6E10 at various concentrations in PBS or KB) was carried out for at least 600 s and followed by a dissociation step for at least 600 s in PBS or KB. Background of pAbs-Aβ or 6E10 binding to unconjugated biosensor tip was also measured and subtracted. The assays were analyzed and fitted with the Octet Software 7.0.1.1 (FortéBio Inc.).

### Thioflavin T aggregation assay

Aggregation of Aβ was monitored by using the Thioflavin T (ThT) binding assay, which identifies amyloid containing β-sheet structures. 5 µM Aβ42 was incubated with test substances diluted accordingly. The assay was performed with 50 µM ThT, 150 mM NaCl, and 10 mM sodium phosphate at 37°C. The fluorescence intensity in each well of a 96-well plate was measured in an EnVision multilabel reader (Perkin Elmer) at 456 nm (Excitation) and 482 nm (Emission), every 15 min for 20 h. Each data point was determined in triplicate and each assay was performed a minimum of 3 times.

### SH-SY5Y cell culture and toxicity studies

SH-SY5Y human neuroblastoma cells were obtained from ATCC (ATCC, St. Cloud, MN) and grown in Dulbecco's modified Eagle's medium (DMEM) supplemented with 10% FCS, 10 mM HEPES, 4 mM glutamine and 10 µM all-trans Retinoic acid at 37°C in a 5% CO_2_ atmosphere. Cells were plated and differentiated for 72 h at a density of 10,000 cells per well in a 96-well microtiter plate. After removal of medium Aβ monomers (10 µM final concentration) were added in 100 µl of fresh medium with appropriate dilutions of test substances and incubated for 72 h. Each data point was determined in hexaplicate. LDH toxicity assay (Sigma, St. Louis, MO) was performed according to the manufacturer's protocol.

### Preparation of primary cortical neurons from rat and immunocytochemistry

Primary rat cortical neuron cultures were performed using embryos at day 18 (E18) from which the neurons were dissected as described previously [25]. Briefly, pregnant rats were killed by CO_2_ inhalation to minimize suffering. The embryos were removed, immediately decapitated and cortical neurons were isolated from the brains. Cultures were incubated for 5 days *in vitro* (DIV) at 37°C and 5% CO_2_. For the assay 10 µM Aβ42 (preparation see above) alone or co-incubated with test substances were added directly to the cultures and incubated for 24 h. All animal use was approved by the Swiss Institute for Animal Welfare and was in compliance with all federal and state regulations (approval ID 1/10, Kantonales Veterinäramt Bern). Animal experiments were kept to a minimum.

For immunocytochemistry the cells were fixed with 4% paraformaldehyde and stained with primary antibodies anti-Map2, 1∶1000 (Millipore, Billerica, MA) and anti-Aβ42, 1∶250 (Merck, Darmstadt, Germany). Incubation with secondary antibodies anti-mouse Cy2, 1∶500, anti-rabbit Cy3, 1∶1000, anti-human IgG Fc Cy2, 1∶100, anti-human IgG DyLight 405, 1∶100 and anti-mouse IgG Dylight 405, 1∶100 (all Jackson Immunoresearch Ltd., West Grove, PA) was followed by image acquisition with a Zeiss LSM 5 Exciter (Zeiss, Jena, Germany).

### BV-2 cell culture and phagocytosis assay

The BV-2 murine microglial cell line was originally developed by Dr. V. Bocchini at the University of Perugia (Perugia, Italy) [26] and kindly provided by Dr. A. Fontana (University Hospital Zurich, Zurich, Switzerland). BV-2 cells were shown to exhibit phenotypic and functional properties comparable to those of primary microglia [26] and are considered as a suitable model for in vitro studies of activated microglial cells [27]. Briefly, BV-2 cells were grown and maintained in DMEM supplemented with 10% FCS.

The phagocytosis assay was performed according to Webster et al. [28]. Briefly, BV-2 cells were seeded in 24-well plates at a density of 100'000 cells/well and left to attach in DMEM with 10% FCS at 37°C/5% CO_2_. After 6 h the medium was replaced by DMEM/F12 and the cells were incubated over night at 37°C/5% CO_2_. 90 min before the experiment the cells were equilibrated with DMEM/F12, 1% BSA. An inhibitor of clathrin-mediated endocytosis (Cytochalasin D; 5 µM), Scavenger Receptor-inhibitor Fucoidan (100 µg/ml) and inhibitor of FcR [29] (Blebbistatin; 100 µM) were added for 30 min. Fibrillar Aβ42 (2 µM), and test substances were diluted in DMEM/F12, added to the cells and incubated for 30 min at 37°C/5% CO_2_. After several washing steps, propidium iodide was added and the cells were subjected to FACS analysis to determine FITC-fluorescence intensity.

For immunocytochemistry the cells were incubated with test substances for 4 h, fixed with 4% paraformaldehyde and stained with primary antibodies anti-LAMP1 (Lysosomal-associated membrane protein 1), 1∶200 (Abcam, Cambridge, UK) and anti-Cleaved Caspase-3, 1∶200 (Cell Signaling, Danvers, MA), anti-CD11b, 1∶100 (Abcam), anti-Rab5, 1∶100 (Abcam), anti-Rab7, 1∶100 (Abcam), anti-Actin, 1∶100 (Abcam). Incubation with secondary antibodies anti-rabbit Cy3, 1∶1000, anti-mouse Cy3, 1∶1000, anti-mouseDyLight405, 1∶100, anti-rat DyLight 649, 1∶100 (Jackson Immunoresearch Ltd.) was followed by image acquisition using a Zeiss LSM 5 Exciter (Zeiss).

### Long-Term Potentiation assay

The assay was performed at QPS Austria (Graz, Austria) using standard procedures. Briefly, 350 µm thick transverse hippocampal slices were prepared from brains of 3 months old mice using a McIlwain tissue chopper (Campden Instruments, Loughborough, UK). Slices were incubated in standard artificial cerebrospinal fluid (aCSF) at ambient temperature for 60 minutes, which were constantly gassed with 95% O_2_–5% CO_2_. aCSF contained 130 mM NaCl, 3.5 mM KCl, 2 mM CaCl_2_, 2 mM MgCl_2_, 0.96 mM NaH_2_PO_4_, 24 mM NaHCO_3_, 10 mM D-glucose (pH 7.4). Individual slices were transferred to a 3D-MEA chip with 60 tip-shaped and 60-µm-high electrodes spaced by 100 µm (Ayanda Biosystems, S.A., Lausanne, Switzerland). The slices were continuously perfused with oxygenated aCSF (1.5 ml/min at 34°C) during the complete recording session. Data were recorded by a standard, commercially available MEA setup (Multi Channel Systems MCS GmbH, Reutlingen, Germany). The Schaffer-collateral was stimulated by injecting a biphasic current waveform (−100/+100 µs) through one selected electrode at 0.033 Hz. The peak-to-peak amplitudes of field excitatory postsynaptic potentials (fEPSPs) at the stratum pyramidale and stratum radiatum of CA1 were recorded. Following a stable 30-min control sequence LTP was induced using a theta-burst stimulation (TBS) pattern applied at the maximum stimulation intensity. TBS comprised four trains administered at 20 s intervals with 10 bursts given at 5 Hz per train and 4 pulses given at 100 Hz per burst. The mice (CFLP, Animal Breeding Facility, University of Szeged) were kept and the experiments were conducted in conformity with Council Directive 86/609/EEC, the Hungarian Act of Animal Care and Experimentation (1998, XXVIII). For the generation of oligomers, Aβ42 was dissolved in oxygenated aCSF at 50 µM and incubated for 24 h at 37°C prior to administration. The Aβ solution was diluted to 1 µM Aβ and mixed with Privigen or Proline at the concentration/dilution of interest. Privigen (100 mg/ml) and Proline stock solution (250 mM, pH 4.8) were further diluted to the appropriate concentration using oxygenated aCSF.

### Statistical analysis

Data were expressed as means ± SD. Statistical analysis was performed by Student's t-test or ANOVA and Bonferroni's Post-hoc test using the GraphPad Prism 5 software (GraphPad Software Inc., La Jolla, CA). A value of p<0.05 was considered statistically significant.

## Results

### Characterization of Aβ preparations

**Figure 1:**
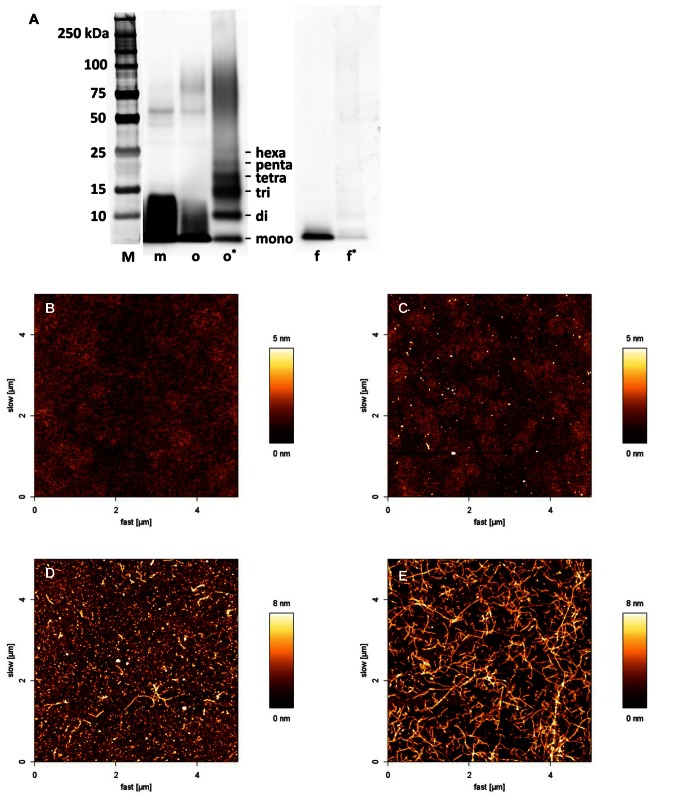
Characterization of Aβ preparations. The Aβ preparations that were used in subsequent assays were characterized by SDS-PAGE (A) and AFM (B–E). **A** Silver Staining of Aβ42 monomers (m), oligomers (o), oligomers cross-linked with Peroxynitrite (o*), fibrils (f) and fibrils cross-linked with peroxynitrite (f*) separated on a 4–12% Bis-tris gel. **B–E** AFM of blank (B), Aβ42 monomers in NaOH 10 mM (C), oligomers in PBS (D), fibrils in HCl 10 mM (E). WB and AFM revealed cross-linked Aβ oligomers ranging in size from dimers to hexamers. Every batch of oligomers was tested accordingly to assure consistent quality for comparable results. **M** Marker.

To ensure consistent quality of the oligomer preparations used, these preparations were continuously controlled by Western Blot analysis and Atomic Force Microscopy (AFM). Figure 1 shows typical results of this characterization. The silver staining (Fig. 1A) showed that the freshly prepared monomeric fraction already contained a small amount of Aβ oligomers (m). The incubation of Aβ monomers for 4 h at 30°C resulted in the formation of different oligomer species (o). However, the cross-linking of oligomers with peroxynitrite resulted in reproducible formation of Aβ oligomers ranging in size from dimers to hexamers with only very few monomers and fibrils (o*). The cross-linked Aβ oligomer preparation was used for the ELISA assays and affinity chromatography. Additionally, two fibril preparations were analyzed. Due to the large size of the fibrils they did not enter the gel and are therefore not visible by silver staining. The cross-linking had only a small effect on the fibrils. It resulted in the stabilization of the fibril and therefore only very few Aβ monomers remained (f*), whereas the proportion of free monomers was much larger in non-cross-linked fibrils (f), since monomers are in constant equilibrium with fibrils.

The formation of Aβ oligomers and fibrils was also monitored by AFM (Fig. 1B–E). As already revealed by the Western Blot analysis, the fresh monomeric preparation also contained some small oligomers (Fig. 1C). AFM of the oligomeric preparation showed larger aggregates and also some small fibrils (Fig. 1D). The fibrillar preparation contained mostly large Aβ fibrils (Fig. 1E).

According to the results obtained by SDS-PAGE and AFM the cross-linked Aβ oligomer preparation used contained mostly Aβ oligomers ranging in size from dimers to hexamers.

### IVIG and pAbs-Aβ specifically bound to Aβ42 oligomers

ELISA assays performed with Privigen revealed that the Aβ-specific antibodies in the IVIG preparation showed the strongest binding to Aβ oligomers and fibrils but a weaker binding to monomers (data not shown). This finding supports the idea that natural antibodies preferentially bind structural epitopes in the Aβ peptide [30], [31]. In accordance with these results, the affinity purification of pAbs-Aβ from Privigen was performed with Aβ oligomers that were coupled to the column resin. The purification yielded pAbs-Aβ in the range of 0.1% of the IVIG concentration.

**Figure 2:**
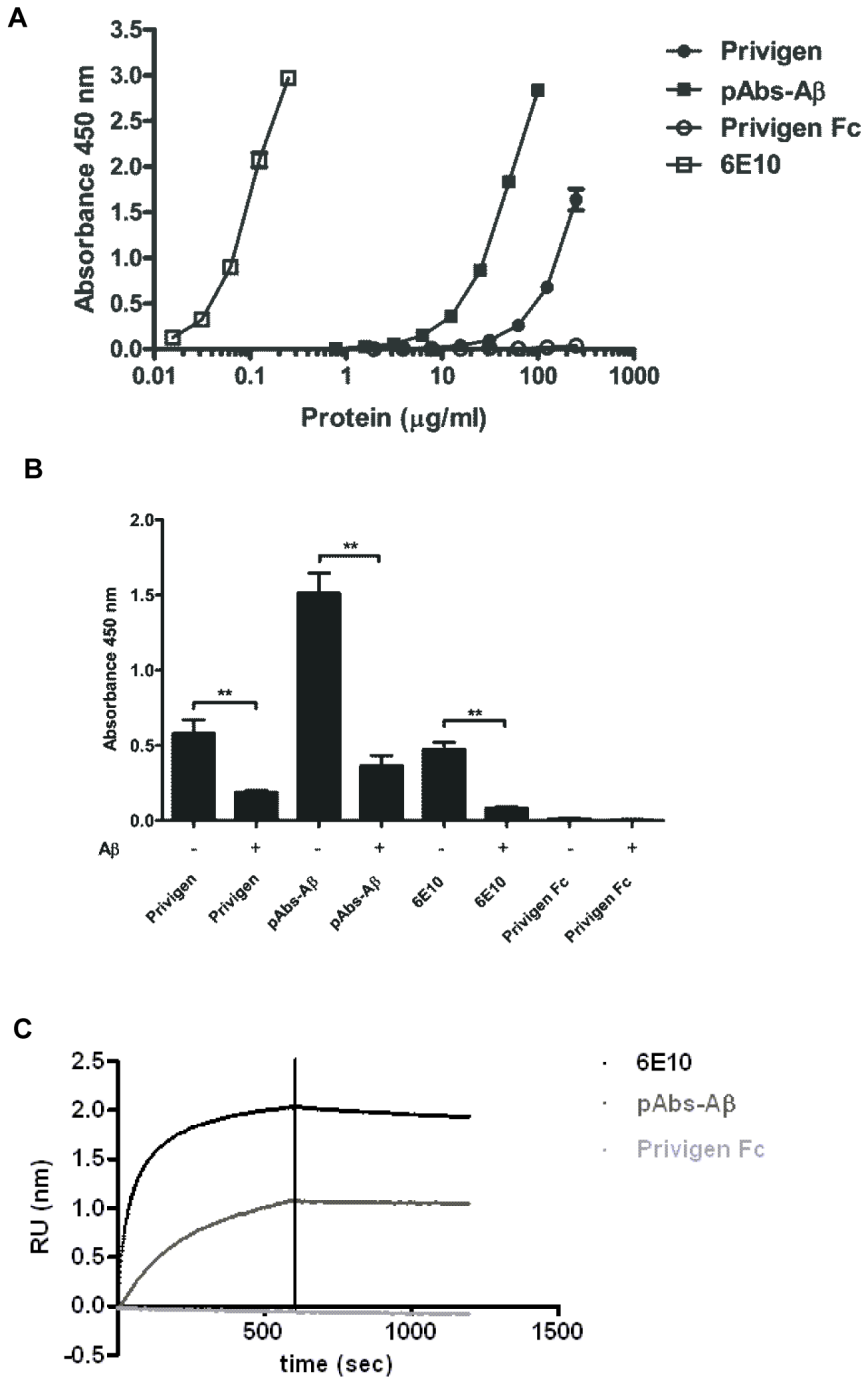
Specific binding of Privigen and pAbs-Aβ to Aβ oligomers. Determination of specific binding to Aβ42 oligomers by ELISA (A and B) and real-time binding by BioLayer Interferometry (C). **A** Privigen, pAbs-Aβ and 6E10 showed different binding activities to the Aβ42 oligomer-coated plate, with 6E10 mAb exhibiting the strongest binding followed by pAbs-Aβ and Privigen. Privigen Fc fragment showed no binding to Aβ42 oligomers. **B** A competition ELISA showed that the binding of Privigen, pAbs-Aβ and 6E10 could be competed by pre-incubation with Aβ42, thereby confirming specificity to Aβ42 oligomers. **C** BioLayer Interferometry (Octet System). Binding curves of pAbs-Aβ, 6E10 and Privigen Fc fragment on monomeric biotinylated Aβ. measured at 133 nM. In an independent experiment, the apparent K_D_ value for the pAbs-Aβ was calculated to be 1.5*10^−7^±1.8*10^−8^ M and 2.1*10^−8^±1.3*10^−9^ M for 6E10. Privigen Fc showed no binding to the Biosensor tip.

ELISA analysis on Aβ oligomer coated plates revealed that pAbs-Aβ showed approximately a 10-fold stronger binding to Aβ oligomers than IVIG as estimated by the ELISA signal. The Aβ-specific control antibody 6E10 exhibited as expected the strongest binding to Aβ oligomers, whereas Privigen Fc fragments showed no binding (Fig. 2A).

A competition ELISA was performed to investigate the specificity of the binding to Aβ oligomers. After pre-incubation with Aβ the ELISA showed significantly reduced signals for Privigen, pAbs-Aβ and 6E10. This result supports the observation that Privigen and pAbs-Aβ specifically bound to Aβ oligomers and that this interaction could be competed by pre-incubation with Aβ.

The binding affinity of the pAbs-Aβ was additionally determined by BioLayer Interferometry (Octet) and compared to the affinity of Aβ-specific monoclonal antibody 6E10. pAbs-Aβ, 6E10 and Privigen Fc fragment (each 167 nM) were measured in parallel and corrected for background. The monoclonal antibody 6E10 showed a higher response and a higher binding affinity to Aβ compared to the pAbs-Aβ. Privigen Fc fragment showed no binding to the Aβ-coated biosensor (Fig. 2C). Additionally we determined the K_D_ values of pAbs-Aβ and 6E10 from dilution series (Fig. S1 in File S1), which were calculated to be 1.5*10^−7^±1.8*10^−8^ M and 2.1*10^−8^±1.3*10^−9^ M, respectively.

Therefore, Privigen contained approximately 0.1% pAbs-Aβ, which bound to Aβ with slightly weaker affinity than 6E10.

### IVIG and pAbs-Aβ inhibited Aβ fibril formation

**Figure 3:**
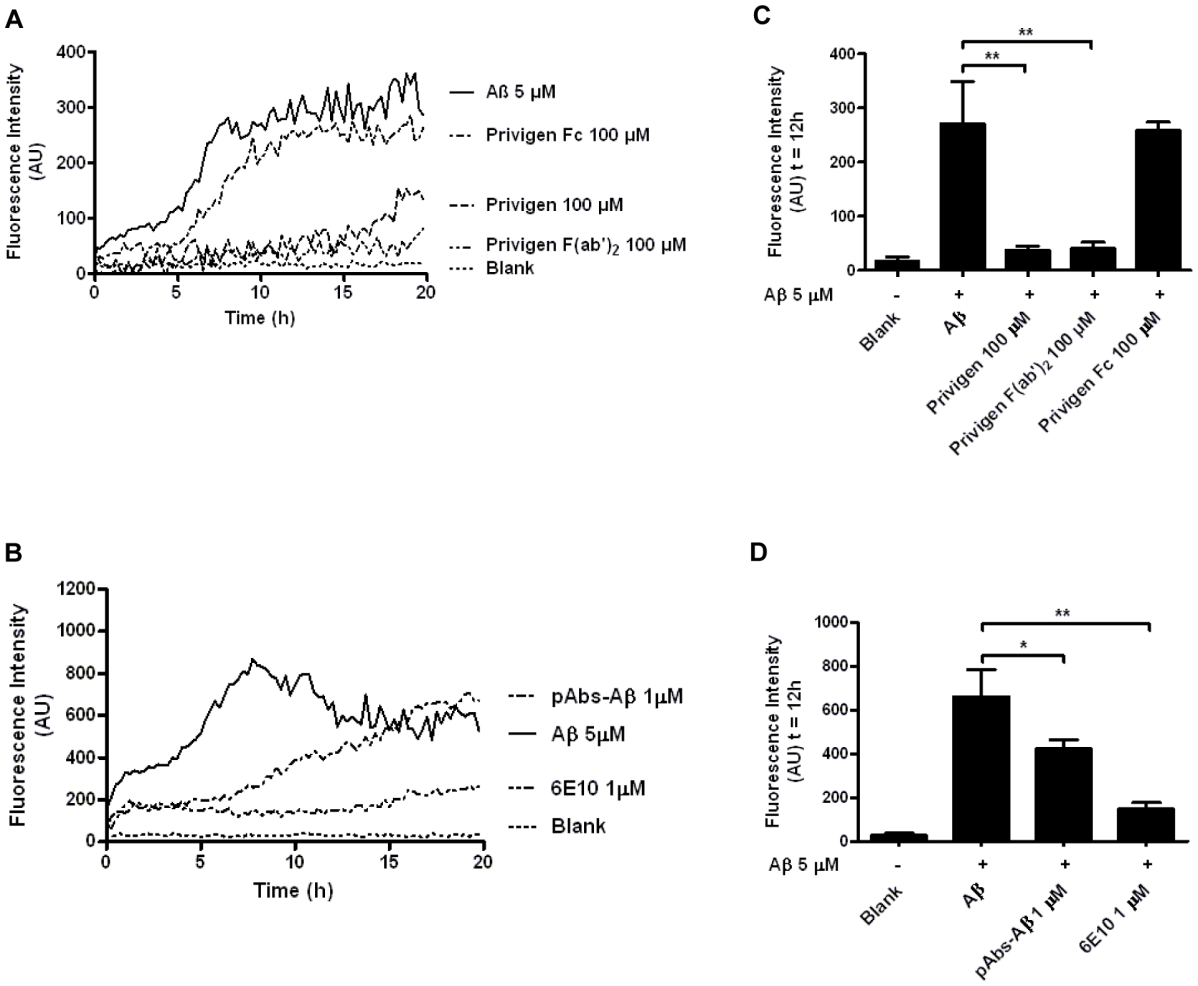
Inhibition of Aβ fibril formation by incubation with Privigen and pAbs-Aβ. Thioflavin T assay showing aggregation kinetics of recombinant Aβ42 (5 µM) alone or co-incubated with either **A** Privigen, Privigen F(ab′)_2_ fragment, and Privigen Fc fragment at 100 µM or with **B** Privigen, pAbs-Aβ and 6E10 at 1 µM. Recombinant Aβ42 was incubated with various compounds at 100 µM **C** or 1 µM **D** and Thioflavin T fluorescence was measured at t = 12 h. Aβ aggregation was significantly decreased by incubation with Privigen and Privigen F(ab)_2_ at 100 µM and pAbs-Aβ and 6E10 at 1 µM but not with Privigen Fc at 100 µM (* p<0.05; ** p<0.005).

The ability of IVIG and pAbs-Aβ to inhibit Aβ aggregation was assessed in an aggregation inhibition assay. Monomeric Aβ42 (5 µM) was incubated either alone or with the addition of Privigen (1 µM, 100 µM), Privigen F(ab′)_2_ fragment (100 µM), Privigen Fc fragment (100 µM), pAbs-Aβ (1 µM) or 6E10 (1 µM) (Fig. 3). Kinetic analysis of Aβ42 incubated with Privigen and Privigen F(ab)_2_ fragment revealed that Aβ aggregation was significantly delayed or even inhibited. Privigen Fc fragment in contrast showed no significant inhibition of aggregation (Fig. 3A). Inhibition of aggregation was dose-dependent for Privigen, and Privigen F(ab′)_2_ fragment and disappeared at 1 µM (Fig. S2 in File S1). Statistical analysis of Thioflavin T fluorescence measured at 12 h of aggregation showed a significant reduction of Thioflavin T fluorescence - and therefore Aβ aggregation - for Privigen and Privigen F(ab′)_2_ fragment at 100 µM (Fig. 3C; * p<0.05; ** p<0.005).

Kinetic analysis of 5 µM Aβ42 incubated with Privigen, pAbs-Aβ and 6E10 (all 1 µM) revealed that pAbs-Aβ delayed Aβ aggregation, Privigen (data not shown) had no effect whereas 6E10 completely inhibited Aβ aggregation (Fig. 3B). When the Thioflavin T fluorescence was measured after 12 h of aggregation, statistical analysis revealed that both pAbs-Aβ and 6E10 significantly reduced Aβ aggregation (Fig. 3D; * p<0.05; ** p<0.005). A dose-response comparison of Privigen and purified pAbs-Aβ revealed that pAbs-Aβ were approximately 100-fold more effective in inhibiting Aβ aggregation than Privigen (Fig. S2 in File S1). This result is in good correlation with the finding that about 0.1% pAbs-Aβ can be purified from Privigen.

### IVIG and pAbs-Aβ rescued SH-SY5Y from Aβ toxicity and prevented the binding of Aβ to neurons

**Figure 4:**
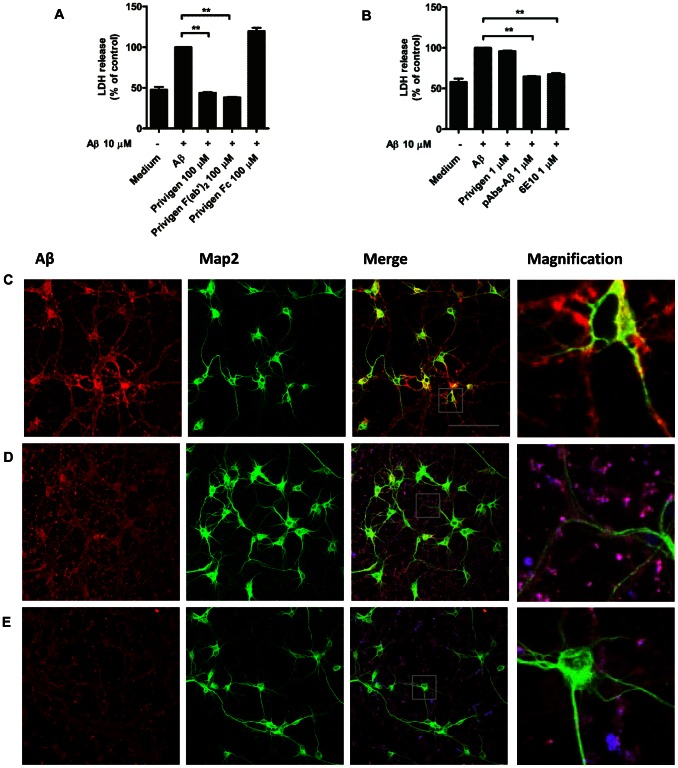
Privigen and pAbs-Aβ prevented Aβ-mediated cytotoxicity in SH-SY5Y cells and inhibited Aβ-binding to primary cortical neurons. **A and B** SH-SY5Y cells were treated with 10 µM monomeric Aβ42 alone or co-incubated with **A** Privigen, Privigen Fc fragment and Privigen F(ab′)_2_ fragment at 100 µM, or with **B** Privigen, pAbs-Aβ and 6E10 at 1 µM. Cell death was measured by LDH release, normalized to Aβ and expressed in percent. Aβ-mediated neurotoxicity was significantly reduced by Privigen and Privigen F(ab′)_2_ fragment at 100 µM (A) and by pAbs-Aβ and 6E10 at 1 µM (B) but not by Privigen Fc fragment at 100 µM (A) and Privigen at 1 µM (B) (** p<0.005). **C to E** Staining of rat primary cortical neurons (DIV5) after treatment with **C** 10 µM monomeric Aβ alone or co-incubated with **D** Privigen (45 µM) or **E** pAbs-Aβ (0.5 µM). Confocal images showed that Privigen at 45 µM and pAbs-Aβ at 0.5 µM apparently disrupted the binding of Aβ to the neuronal cell body and dendrites, which resulted in the distribution of Aβ in the medium as immune-complexes. Microtubule-associated protein 2 (green), Aβ42 (red), antibody test substances (blue), immune-complexes (violet). Bar 100 µm.

Since Aβ is toxic to neurons, the effect of IVIG and pAbs-Aβ on Aβ-mediated toxicity was evaluated. SH-SY5Y cells were incubated with 10 µM monomeric Aβ42 alone or co-incubated with Privigen, Privigen F(ab′)_2_ fragment, Privigen Fc fragment, pAbs-Aβ or 6E10. Aβ-mediated neurotoxicity was significantly reduced by Privigen (100 µM) and Privigen F(ab)_2_ fragment (100 µM) (Fig. 4A; ** p<0.005), pAbs-Aβ (1 µM) and 6E10 (1 µM) (Fig. 4B; ** p<0.005) but not by Privigen Fc fragment (100 µM) nor by Privigen at 1 µM.

The results of the toxicity assays were further supported by immunostaining of rat primary cortical neurons which had been treated with 10 µM monomeric Aβ alone (Fig. 4C) or co-incubated with Privigen (45 µM) (Fig. 4D), Privigen Fc fragment (45 µM) (Fig. S3 in File S1), pAbs-Aβ (0.5 µM) (Fig. 4E) or 6E10 (0.5 µM) (Fig. S3 in File S1). Confocal imaging revealed that neurons treated with Aβ alone were surrounded by Aβ aggregates which resulted in shortened or degenerated neuritic processes and swollen cell bodies. Incubation with Privigen at 45 µM, 6E10 at 0.5 µM and pAbs-Aβ at 0.5 µM inhibited the binding of Aβ to the neuronal cell body and the dendrites, which resulted in the distribution of Aβ in the medium as immune-complexes. These neurons exhibited more intact neuronal morphology with longer processes than conditions where the cells were treated with Aβ42 alone or co-incubated with Privigen Fc fragment. In these conditions Aβ remained bound to the neurons and dendrites, which further supports the findings of the toxicity assay.

These data suggest that Privigen and pAbs-Aβ protect neurons from Aβ-induced toxicity by inhibition of Aβ binding to neuronal processes.

### IVIG increased phagocytosis of fibrillar Aβ

**Figure 5:**
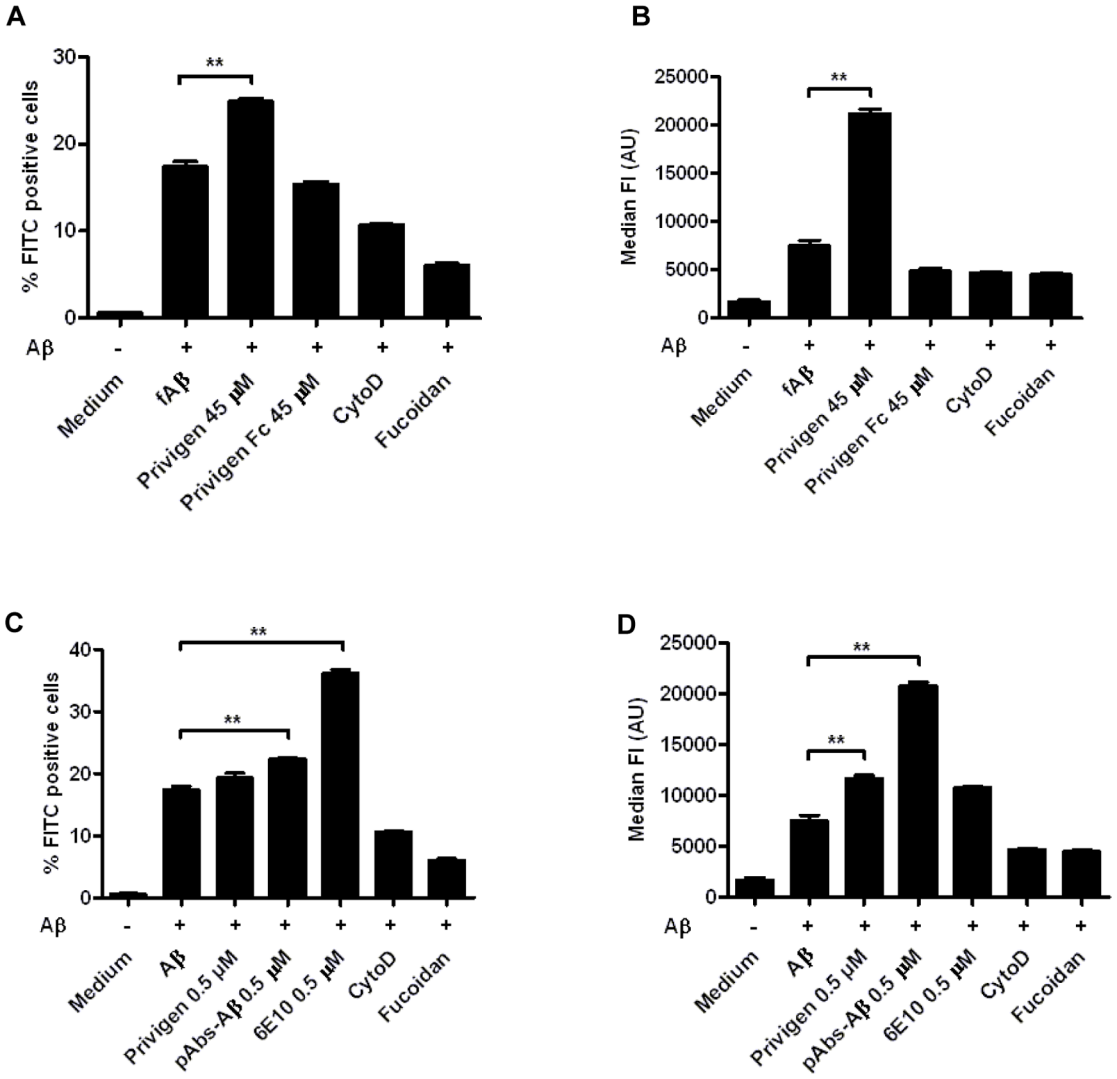
Privigen and pAbs-Aβ increased the phagocytosis of fibrillar Aβ. BV-2 cells were treated for 30 min with 2 µM FITC-labelled Aβ fibrils alone or co-incubated with Privigen and Privigen Fc fragment at 45 µM, **A and B** or with Privigen, pAbs-Aβ and 6E10 at 0.5 µM, **C and D**. Cytochalasin D was added as an inhibitor for receptor-mediated endocytosis and fucoidan as an inhibitor of scavenger receptor A/B. At 45 µM only Privigen significantly increased the number of phagocytosing cells (A) and the amount of phagocytosed fibrillar Aβ in the FITC-positive cell population (B). At 0.5 µM Privigen slightly whereas pAbs-Aβ and 6E10 significantly increased the number of FITC-positive cells (C). Only with Privigen and pAbs-Aβ the amount of phagocytosed Aβ was significantly increased, whereas 6E10 resulted only in a slightly not statistically significant increased phagocytosis of fibrillar Aβ (D) (** p<0.005).

Aβ activates microglia and in the brains of AD patients amyloid plaques are surrounded by activated microglia [32]. These microglia contain intracellular Aβ indicating that microglia take up fibrillar material [33]. To assess our hypothesis that IVIG and pAbs-Aβ modulate microglial phagocytosis we evaluated the effects of these immunoglobulin preparations on the uptake of FITC-labeled fibrillar Aβ by BV-2 microglia. The incubation of BV-2 cells with 2 µM FITC-labeled fibrillar Aβ and Privigen at 45 µM (Fig. 5A), pAbs-Aβ at 0.5 µM and 6E10 at 0.5 µM resulted in a significantly increased number of positive cells when compared to Aβ alone but only slightly increased by Privigen at 0.5 µM (Fig. 5C) (** p<0.005).

Notably, the amount of phagocytosed fibrillar Aβ in the FITC-postive cell population (Fig. 5B, and D) was significantly increased by incubation with Privigen at 45 µM and 0.5 µM as well as by pAbs-Aβ at 0.5 µM (** p<0.005). In contrast, incubation with 6E10 resulted only in a slight increase in the amount of ingested fibrillar Aβ (Fig. 5D). Cytochalasin D, an inhibitor for clathrin-mediated endocytosis, and Fucoidan, an inhibitor of scavenger receptors A and B, effectively inhibited phagocytosis of fibrillar Aβ alone, indicating that receptor-mediated endocytosis and specifically that Scavenger Receptors A and/or B (SR A/B) are involved in the phagocytosis of fibrillar Aβ by BV-2 cells. When fibrillar Aβ was co-incubated with Privigen and Cytochalasin D there was a complete inhibition of phagocytosis (data not shown). In contrast, co-incubation of fibrillar Aβ with Privigen and Fucoidan did not prevent phagocytosis (data not shown). This indicates that phagocytosis of immune-complexes consisting of fibrillar Aβ and Privigen are probably taken up by microglia via Fc receptors (FcR) in addition to SR A/B.

**Figure 6:**
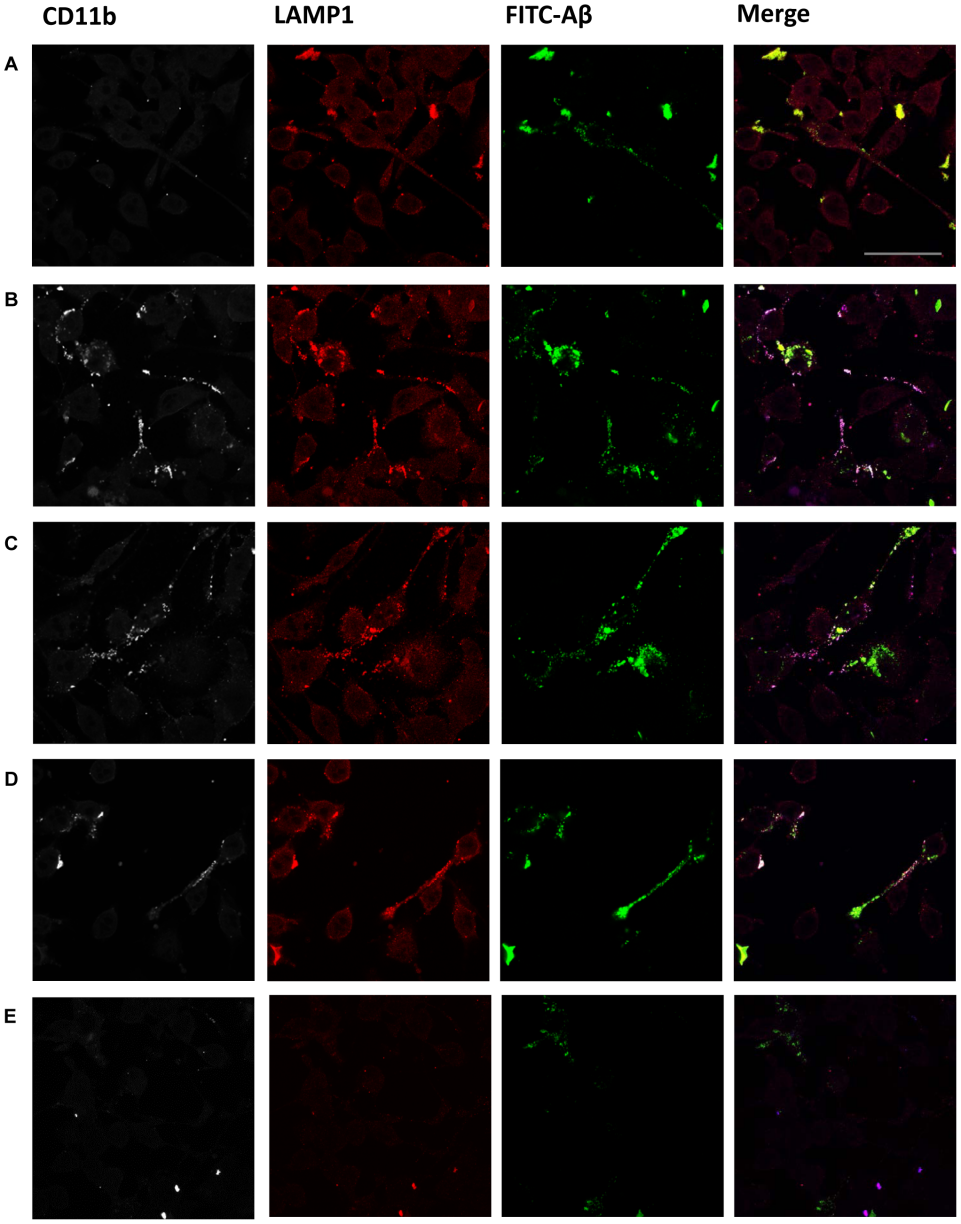
Privigen and pAbs-Aβ increased CD11b expression on BV-2 cells and internalized Aβ co-localized with lysosomes. BV-2 cells were treated for 4 h with **A** 2 µM FITC-labelled Aβ fibrils alone or co-incubated with **B** Privigen (45 µM) and **C** pAbs-Aβ (0.5 µM), **D** Privigen (45 µM) and Fucoidan or **E** Privigen (45 µM)and Blebbistatin. Incubation with Privigen and pAbs-Aβ resulted in increased uptake of FITC-Aβ fibrils and upregulation of CD11b expression Co-incubation with Privigen and Fucoidan did not abolish Aβ phagocytosis and upregulation of CD11b was still detectable. Incubation of Aβ alone or with Fucoidan showed weak uptake of FITC-Aβ into microglia but without upregulation of CD11b expression. The intracellular FITC-Aβ signal was localized in punctate structures that co-localized with CD11b immunoreactivity and LAMP1 immunoreactivity in cells incubated with Privigen and pAbs-Aβ. CD11b (white), LAMP1 (red), FITC-Aβ (green). Bar 50 µm.

Microscopic analysis of the BV-2 microglia revealed that after 4 h of phagocytosis Aβ was present inside the cells in punctate structures (Fig. 6A). When fibrillar Aβ was co-incubated with Privigen at 45 µM (Fig. 6B), pAbs-Aβ at 0.5 µM (Fig. 6C) and 6E10 at 0.5 µM (data not shown) the amount of internalized Aβ was increased. However, these structures were reduced or absent when incubated with Privigen Fc at 45 µM, Cytochalasin D or Fucoidan (Fig. S4 in File S1), with large Aβ aggregates remaining outside the cells. Interestingly, the increased phagocytic activity observed when Aβ was co-incubated with Privigen at 45 µM (Fig. 6B), Privigen and Fucoidan (Fig. 6D), pAbs-Aβ (Fig. 6C) and 6E10 (data not shown) was accompanied by increased immunoreactivity for CD11b. No activation of microglia as indicated by CD11b expression was observed when Aβ was incubated alone (Fig. 6A), co-incubated with Privigen Fc fragment (Fig. S4 in File S1) or with Privigen and Cytochalasin D (data not shown), indicating that Aβ has to be internalized via receptor-mediated endocytosis and most propably via FcγR-mediated endocytosis to promote CD11b expression. Indeed, inhibition of the FcγR by incubation with Blebbistatin markedly reduced Privigen-mediated endocytosis of fibrillar Aβ and also prevented the expression of CD11b on the cell surface of BV-2 microglia (Fig. 6E). This confirmed the idea that internalization of Aβ immune-complexed with Privigen occurred primarily via FcγR and that this FcγR-mediated endocytosis caused the upregulation of CD11b expression. Furthermore, the internalized FITC- Aβ showed a clear co-localization with lysosomes when incubated with Privigen at 45 µM (Fig. 6B), Privigen and Fucoidan (Fig. 6D), pAbs-Aβ (Fig. 6C) and 6E10 (data not shown).

These data show that Privigen and pAbs-Ab increase the uptake of fibrillar Abta by microglia most probably as immune complexes via the Fcγ receptor (FcγR) in addition to SR A/B. The internalization of immune complexes via FcR leads to anti-inflammatory activation of microglia, which is accompanied by an upregulation of CD11b. This in turn is linked to increased phagocytic activity of microglia and reduced production of ROS.

### Privigen reversed Aβ-mediated LTP inhibition

**Figure 7:**
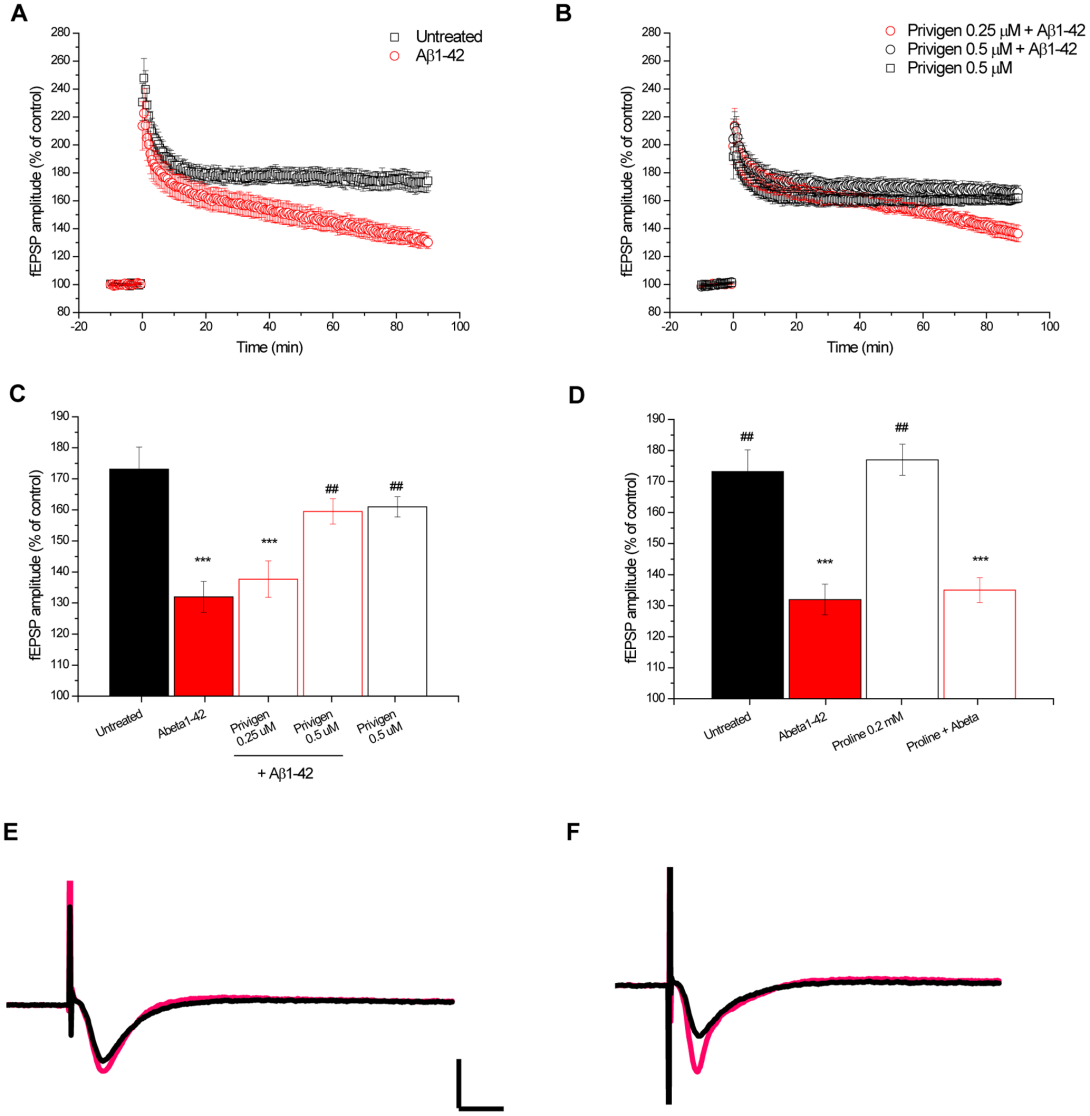
Privigen reversed Aβ-mediated LTP inhibition. LTP assay using mouse brain sections showed that **A** oligomeric Aβ42 caused an impairment of LTP, untreated slices exhibited normal LTP. **B** Privigen protected against Aβ42 when applied at a concentration of 0.5 µM. Privigen at 0.25 µM only slightly improved LTP inhibition. Applied alone at 0.5 µM Privigen had no effect on LTP. **C** Summary of the LTP data 90 min after TBS. ***P≤0.001; compared to Untreated; ##P≤0.01 compared to Aβ42. **D** Summary of the LTP data 90 min after TBS. Proline did not impair LTP and did not modify the effect of Aβ42 on LTP. ***P≤0.001; compared to Untreated; ##P≤0.01 compared to Aβ42. **E and F** Representative fEPSP traces showing evoked responses before (black) and 90 minutes after TBS stimulation (red) for **E** Aβ42 treated slices with fEPSP 90 min after TBS (red) almost as large as before TBS (black) and **F** Privigen (0.5 µM)+Aβ42 treated slices clearly showing that Privigen rescued the Aβ-mediated LTP impairment, since the evoked response after TBS (red) is nearly twice as large as the fEPSP before TBS (black). Calibration bars are 0.5 mV and 15 ms. The number of slices was n = 6 for each group.

Long-term potentiation (LTP) is widely considered one of the major cellular mechanisms that underlies learning and memory [34], [35]. It has also been published that the binding of toxic Aβ oligomers to cell surface receptors on neurons mediated LTP inhibition [36]. Therefore, we investigated the effect of Privigen on LTP. Recordings of fEPSP in mouse hippocampal slices showed inhibition of LTP upon lesion with Aβ oligomers (Fig. 7A). This inhibition was completely reversed by Privigen at 0.5 µM but not at 0.25 µM (Fig. 7B), indicating a clear dose-response. Statistical analysis revealed that treatment with Aβ oligomers significantly inhibited LTP when compared to untreated (***p≤0.001), LTP was still significantly inhibited when treated with Privigen at 0.25 µM (***p≤0.001). LTP inhibition was significantly improved by Privigen at 0.5 µM when compared to Aβ treated slices (##p≤0.01). Privigen alone had no effect on LTP (Fig. 7C). It was also confirmed that the vehicle control proline had no effect on Aβ-mediated LTP inhibition and did not have an effect on LTP itself (Fig. 7D).

These results indicate that Privigen might have the potential to reverse the negative effects of Aβ on synaptic plasticity and might therefore have beneficial effects on cognition in AD patients.

## Discussion

Intravenous Immunoglobulins (IVIG) are manufactured from plasma of healthy donors and have been safely used for more than 30 years for the treatment of a variety of immunological and inflammatory diseases due to their anti-inflammatory and immunomodulatory properties [11], [37], [38]. In two independent preliminary trials the treatment of AD patients with IVIG was associated with decreased CSF Aβ levels, increased plasma Aβ levels and a stabilization or even improvement of cognitive decline in several cases [7], [8], [10]. Furthermore, a retrospective case-controlled analysis found a significantly decreased incidence rate of dementia in patients treated with IVIG [39] compared to age matched untreated controls. Due to these initial promising results two large Phase III clinical trials were initiated (Baxter; NCT00818662; NCT01524887) and just recently a Phase II dose-finding study (Octapharma; NCT00812565) [40] was completed. The data reported significantly increased plasma Aβ40 levels in the 0.4 g/kg every 2 weeks treatment group compared to placebo group [9]. Despite evidence of changes in the distribution of Aβ40 levels the mechanism of action of IVIG in AD is still under debate. We therefore aimed to investigate and compare the efficacy of IVIG and of purified polyclonal Aβ-specific antibodies (pAbs-Aβ) *in vitro*.

In the present study we purified pAbs-Aβ from the IVIG preparation Privigen by affinity chromatography using an Aβ oligomer-coupled resin. We decided to use Aβ oligomers, as Privigen and the pAbs-Aβ preferably bound to structural epitopes found in oligomers and fibrils. This is in agreement with previously published studies showing that natural antibodies predominantly recognize generic structural epitopes present in toxic protein aggregates such as Aβ oligomers and fibrils [16], [30]. Recent studies have implicated Aβ oligomers in the disease mechanism of AD. The degree of synaptic failure and neuronal death in the brain of AD patients and transgenic mice correlates better with the occurrence of soluble Aβ species such as oligomers rather than with amyloid plaques [41], [42].

Analysis of the affinity purified fraction revealed that Privigen contained pAbs-Aβ in the range of 0.1%. This is in good agreement with previous studies [43] showing a similar proportion of natural anti- Aβ antibodies in IVIG preparations prepared from large plasma pools. Evaluation of the binding properties of IVIG and of the purified pAbs-Aβ in an ELISA assay revealed that both showed robust binding to Aβ oligomers but binding of pAbs-Aβ to Aβ oligomers was approximately 10-fold stronger than binding of IVIG. A competition ELISA also confirmed that the binding of pAbs- Aβ and Privigen to Aβ oligomers was specific whereas Privigen Fc fragments showed no binding to Aβ42, confirming that the binding of Privigen and pAbs-Aβ to Aβ42 is dependent on the F(ab)_2_ fragment.

Further analysis of Privigen and pAbs-Aβ showed protection of SH-SY5Y neuroblastoma cells and rat primary cortical neurons from Aβ-mediated neurotoxicity most probably by preventing binding of Aβ to rat primary cortical neurons as shown by confocal microscopy. Several cell surface receptors, such as NMDA receptor [44] or the Prion protein (PrPc) [45] have been implicated as binding partners for Aβ. It has been shown that neurotoxicity is mediated by Aβ binding to receptors on the cell surface and that this binding is sufficient and required to mediate toxicity [46] and inhibition of long-term potentiation (LTP) [36]. A prevention of Aβ binding to neurons as shown with Privigen and the purified pAbs-Aβ could either be achieved by blocking the binding site in the toxic Aβ species or by blocking the receptor on the cell surface. Considering that the purified pAbs-Aβ contain only Aβ-specific antibodies it can be assumed that the observed effect is mainly mediated by blocking the binding site of the peptide and by inhibiting the formation of toxic oligomer species. However, Privigen is a polyclonal mixture of antibodies and also contains antibodies against PrPc, Tau and pTau (Schaub et al., Neurodegenerative Dis. 2011; 7(4) Supplement, Page S670), to name just a few disease relevant targets. Therefore it is possible that, in addition to direct binding of Aβ by Aβ-specific antibodies, the binding to neurons was prevented by blocking the binding partner (e.g. receptor) on the cell surface. This has recently been reported for PrPc [47] whereby blocking the binding of Aβ to PrPc on the cell surface of neurons also abrogated Aβ-mediated toxicity.

A common feature of the AD brain is a reactive gliosis with activated astrocytes and microglia. This neuroinflammatory component of AD is further characterized by release of inflammatory mediators, such as IL-1, MCP-1, MIP-1α, TNFα and S100β, activation of the complement cascade, induction of cyclooxygenase-2 (COX-2) and inducible nitric oxide synthase (iNOS), and production of ROS [12]. This pro-inflammatory reactivity of microglia is known to mediate neurotoxicity and is associated with decreased phagocytic activity of the microglia. It is thought that microglia become chronically activated in AD, which then shifts their activation state towards pro-inflammatory. But, activated microglia have also been found to be intimately associated with neuritic plaques [32] and microglia containing amyloid-β peptide have been detected in AD brains [33]. Activated microglia were thus implicated in active phagocytosis of Aβ as a means of counterbalancing Aβ deposition in the brain parenchyma [48]. Furthermore, it was reported that microglia of AD patients ineffectively phagocytose Aβ [49], probably because they are in a pro-inflammatory activation state. Interestingly, it was shown that immunotherapy with anti-Aβ antibodies [50] or IVIG [13] increased microglial phagocytosis of fibrillar Aβ. We now report that both Privigen and pAbs-Aβ effectively increased the number of phagocytosing BV-2 cells and the amount of phagocytosed Aβ. This effect was blocked by Cytochalasin D and Fucoidan, indicating that fibrillar Aβ is taken up into the cell by clathrin- and Scavenger Receptor (SR) A/B-dependent mechanisms [28]. However, when fibrillar Aβ was co-incubated with Privigen and either Cytochalasin D or Fucoidan, only Cytochalasin D completely inhibited phagocytosis of Aβ. Inhibition of SR A/B only partially prevented phagocytosis of immune-complexed fibrillar Aβ. In contrast, inhibition of FcγR markedly reduced internalization of immune-complexed fibrillar Aβ, which indicates that phagocytosis of immune-complexes also occurred most probably via the FcγR [51], whereas fibrillar Aβ alone is primarily taken up via SR A/B [52]. Microscopic analysis of the BV-2 microglia revealed that Aβ was present inside the cells in punctate structures when fibrillar Aβ was co-incubated with Privigen, pAbs-Aβ and 6E10. In addition to the expected co-localization of FITC- Aβ with lysosomes, we found that increased phagocytosis due to co-incubation with Privigen pAbs-Aβ or 6E10 was accompanied by an upregulation of CD11b. This is indicative of anti-inflammatory microglial activation and has been found to be linked to increased phagocytic activity and reduced production of ROS [53] and might therefore be neuroprotective. Upregulation of CD11b still occurred after inhibition of SR A/B but was completely abolished when FcγR was inhibited. These findings are consistent with previous findings that SR A/B does participate in binding and endocytosis of Aβ fibrils but does not participate in stimulation of intracellular signaling [29], whereas the FcγR has been shown to signal to the nucleus via Syk and Rac [29] thereby possibly upregulating the expression of CD11b. The shift in microglial activation towards an anti-inflammatory activation state by IVIG has also been found in a recent in vivo approach, in which APPswe/PS1dE9 mice were treated for 8 months with IVIG [54]. The authors reported that the treatment had no effect on amyloid plaques, but resulted in increased soluble Aβ levels in the brains of treated mice, which might result in increased neurotoxicity and decreased cognitive performance. Possibly, IVIG was not potent enough to inhibit the amyloid deposition in this aggressive mouse model of brain amyloidosis, which questions the validity of in vivo data generated in mouse models of brain amyloidosis. However, the authors also found increased levels of Iba1-positive activated microglia, but decreased expression of CD45, TNFα and IL-1β. The authors concluded that IVIG treatment caused a shift in the microglial activation state that counterbalanced the negative effects on the brain immune system caused by normal aging. Although it is not clear to what extend in vitro data can be reproduced in vivo, our findings in BV-2 cells concur with in vitro and in vivo results reported by Puli et al. and others [13], [14].Several potential mechanisms have been suggested to be responsible for the action of IVIG in AD patients. Hypotheses can be distinguished by either the action of their naturally occurring polyclonal auto-antibodies against Aβ (pAbs-Aβ) or immunomodulatory effects dependent on IgG [11]. These Aβ –specific antibodies have been shown to modulate Aβ –aggregation and –toxicity [55] as well as microglial activation and phagocytosis [13]. Other AD-relevant auto-antibodies might inhibit polymerization and aggregation of tau, similar to Aβ; and potentially block RAGE and PrPc as cell surface receptors for Aβ [11]. This blockade might inhibit receptor-mediated transport of Aβ across the blood-brain-barrier [11] or prevent cytotoxicity [47], respectively. Independent of a specific antibody-mediated action, IVIG might exert immunomodulatory actions, such as inhibition of complement deposition in or close to Aβ plaques [56], thereby inhibiting activation of the pro-inflammatory complement cascade.

In the presented study we report that the IVIG preparation Privigen contained approximately 0.1% pAbs-Aβ and that both IVIG and pAbs-Aβ bound specifically to Aβ oligomers, inhibited Aβ-aggregation and Aβ-mediated cytotoxicity. The purified pAbs-Aβ exhibited an approximately 10-fold increased affinity to Aβ when compared to the IVIG they were purified from. Furthermore, Privigen and the pAbs-Aβ inhibited the binding of Aβ to primary cortical neurons, which improved neuronal viability and morphology. In addition, both Privigen and pAbs-Aβ increased the number of phagocytosing BV-2 cells as well as the amount of phagocytosed fibrillar Aβ and induced an increase in microglial CD11b expression. Furthermore, we could show that Privigen reversed Aβ-mediated LTP inhibition indeed indicating that IVIG might have a protective effect against Aβ mediated synaptic dysfunction, which may be the basis for memory loss in Alzheimer's Disease [57].

Our results as well as results from others [14] suggest that both the whole fraction of IVIG and purified natural anti-Aβ antibodies may have an impact on Aβ-driven processes in the pathogenesis of AD. AD is a multifactorial disease which will most probably require a multifaceted therapeutic approach. In addition to the effects mediated by specific antibodies the well-established anti-inflammatory and immuno-modulatory properties of IVIG might further add to the beneficial effects observed in clinical studies. Therefore, targeting multiple processes in AD pathogenesis by using IVIG preparations that contain a wide variety of autoreactive natural antibodies might be an attractive approach to improve disease outcome in AD patients

## Supporting Information

File S1 Supporting Information Figures. Figure S1: Dilution series of pAbs-Aβ and 6E10 measured by Octet. Determination of binding affinity of A pAbs-Aβ and B 6E10 to monomeric biotinylated Aβ by BioLayer Interferometry. Apparent K_D_ values were calculated from the dilution series. Figure S2: Dose-response comparison of Privigen and pAbs-Aβ in Thioflavin T assay. Thioflavin T assay showing aggregation kinetics of recombinant Aβ42 (5 µM) alone or co-incubated with either A Privigen at 100 µM, 10 µM and 1 µM or B with pAbs-Aβ at 1 µM, 0.5 µM, 0.1 µM and 0.01 µM. Aβ aggregation was delayed by incubation with Privigen at 100 µM and pAbs-Aβ at 1 µM. Privigen showed no efficacy at 1 µM, pAbs-Aβ lost efficacy at 0.01 µM, indicating that pAbs-Aβ are 100-fold more effective in the Thioflavin T assay than the full IVIG preparation. Figure S3: 6E10 inhibited Aβ-binding to primary cortical neurons but Privigen Fc fragment had no such effect. Staining of rat primary cortical neurons (DIV5) after treatment with 10 µM monomeric Aβ42 and A Privigen Fc fragment at 45 µM or B with 6E10 at 0.5 µM. Confocal images showed that 6E10 at 0.5 µM apparently disrupted the binding of Aβ to the neuronal cell body and dendrites, which resulted in the distribution of Aβ in the medium as immune-complexes. In contrast, incubation with Privigen Fc fragment did not result in the formation of immune-complexes and did not prevent binding of Aβ to the neurons. Microtubule-associated protein 2 (green), Aβ42 (red), antibody test substances (blue), immune-complexes (violet). Bar 100 µm. Figure S4: Blockade of microglial endocytosis of fibrillar Aβ prevents upregulation of CD11b. BV-2 cells were treated for 4 h with 2 µM FITC-labeled Aβ fibrils alone or co-incubated with A Cytochalasin D, B Fucoidan, C Privigen Fc fragment (45 µM) or D Privigen and Cytochalasin D. Incubation with Cytochalasin D, Fucoidan, Privigen Fc fragment and Privigen and Cytochalasin D resulted in markedly reduced uptake of FITC-Aβ fibrils with large Aβ aggregates remaining outside the cells. Also, no upregulation of CD11b expression could be detected when clathrin- and FcγR-mediated endocytosis were blocked. CD11b (white), LAMP1 (red), FITC-Aβ (green). Bar 50 µm.
(PDF)
