# Supplementary material for: Intravenous Immunglobulin Binds Beta Amyloid and Modifies Its Aggregation, Neurotoxicity and Microglial Phagocytosis In Vitro
Source: PLoS One. 2013 May 16;8(5):e63162. doi: 10.1371/journal.pone.0063162 (PMC3656042; doi:10.1371/journal.pone.0063162)

Supplementary Figure S1

A

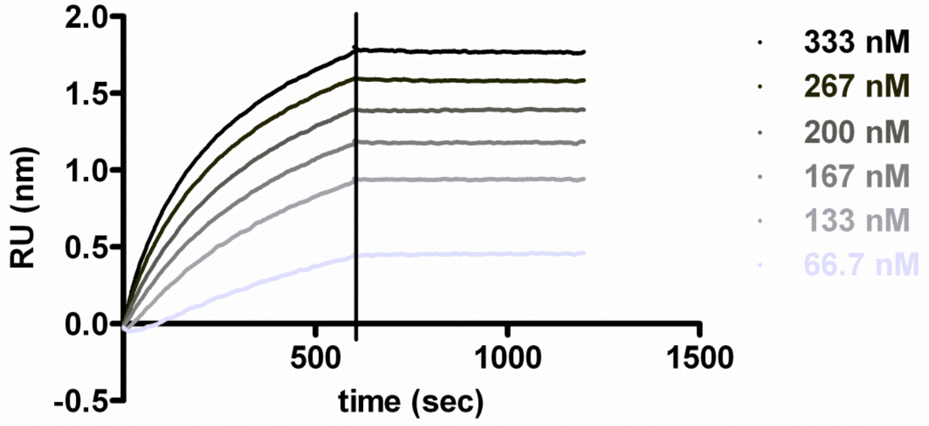

B

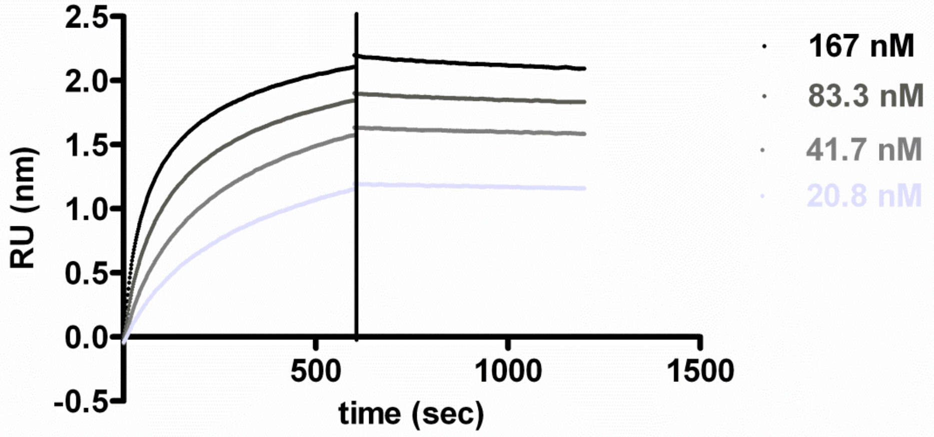

Supplementary Figure S2

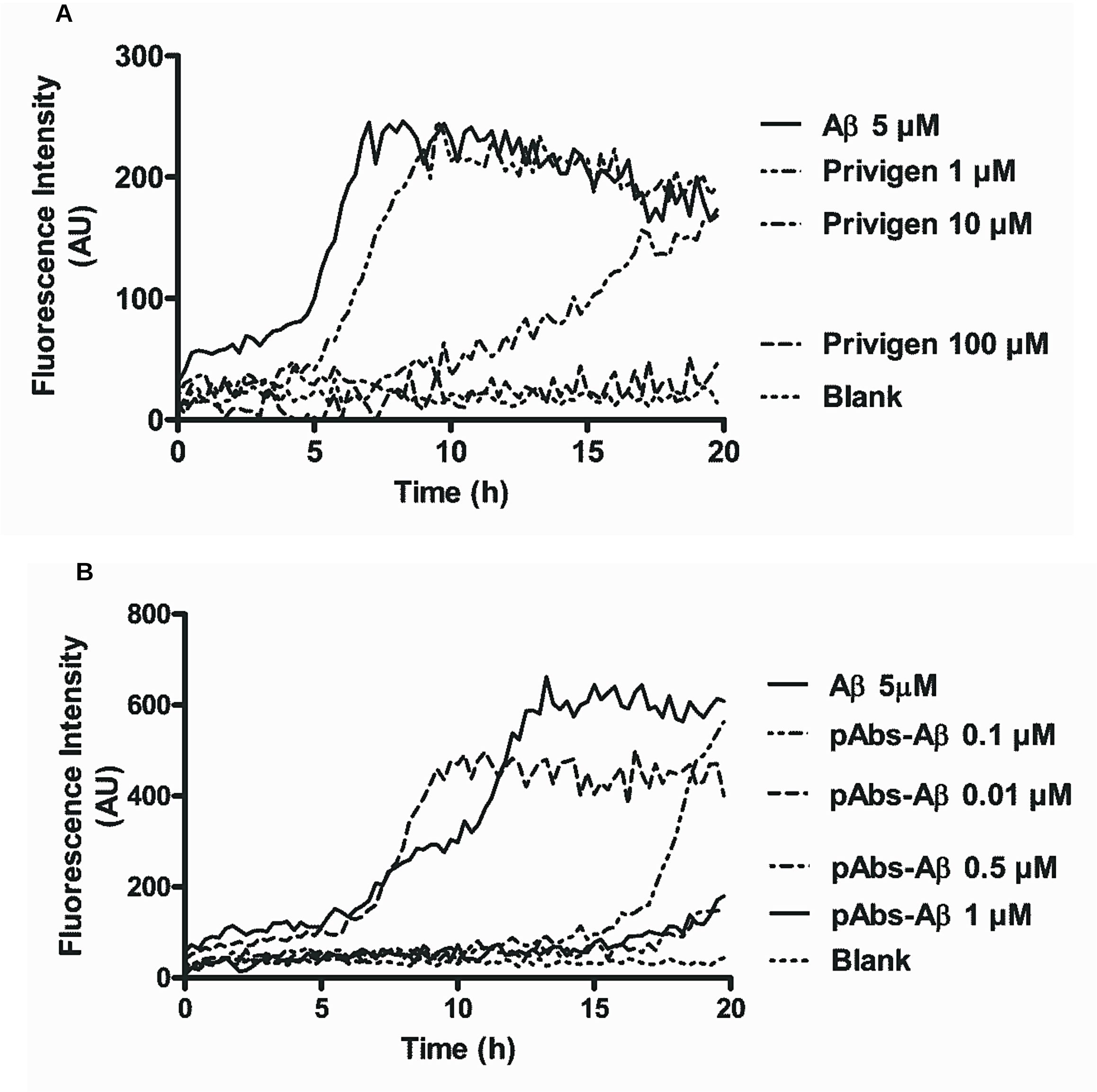

Supplementary Figure S3

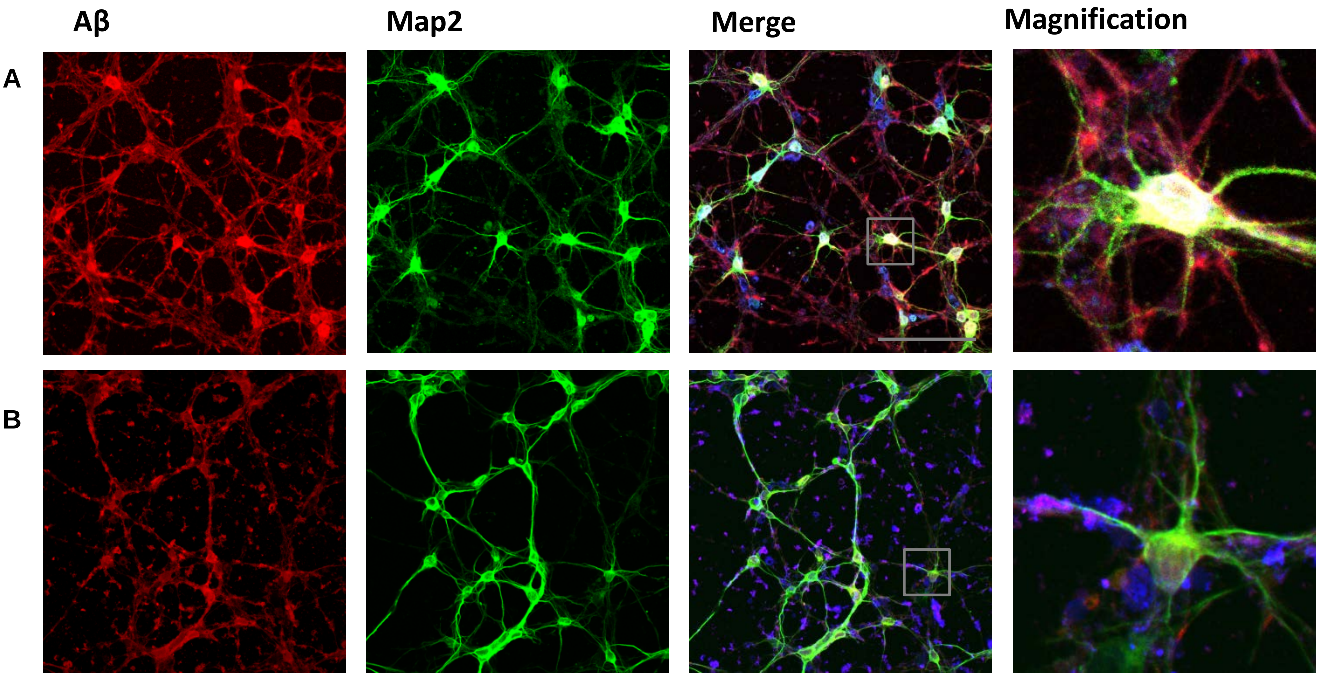

Supplementary Figure S4

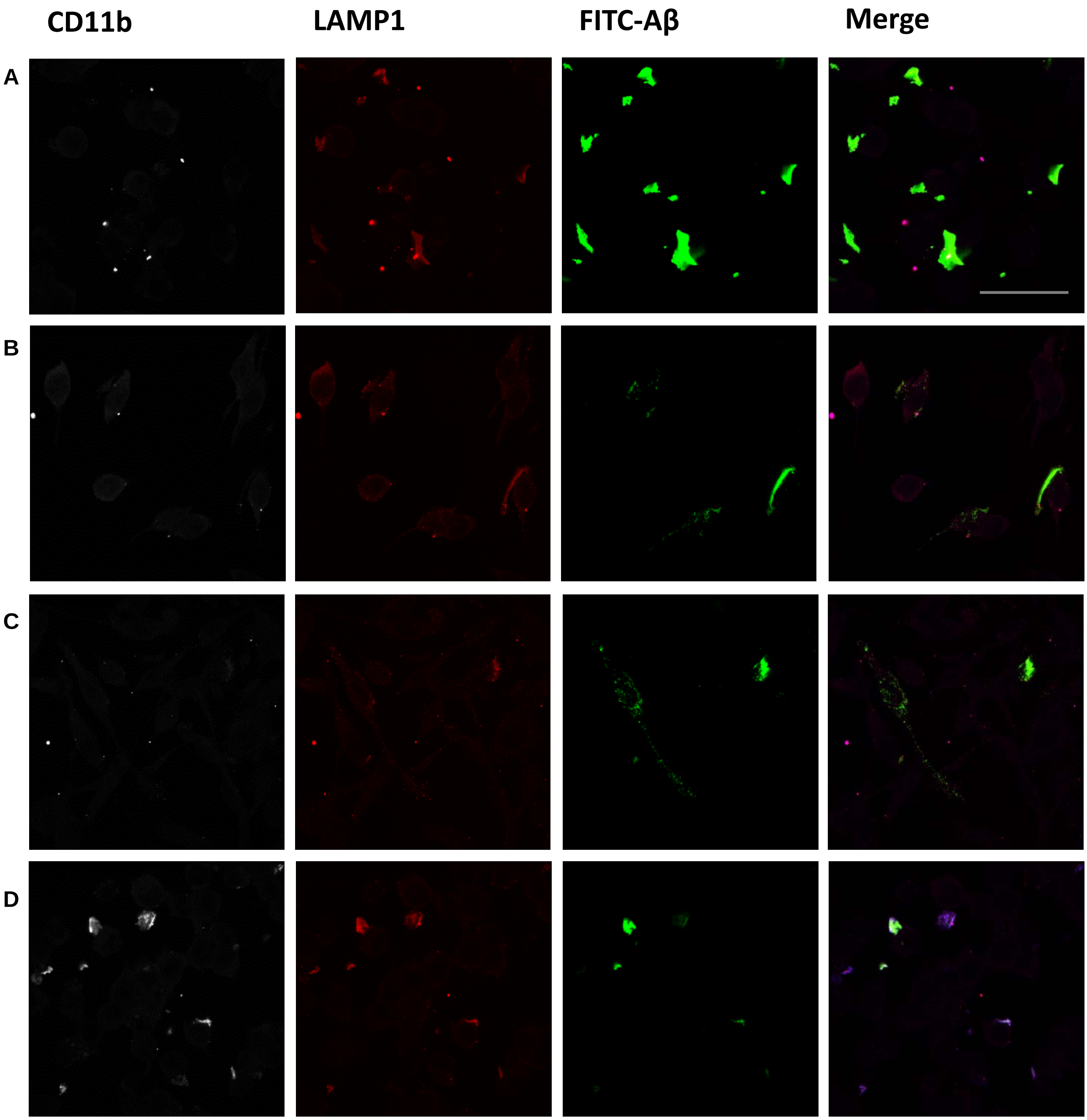

Supplement: File S1 — Supporting Information Figures. Figure S1: Dilution series of pAbs-Aβ and 6E10 measured by Octet. Determination of binding affinity of A pAbs-Aβ and B 6E10 to monomeric biotinylated Aβ by BioLayer Interferometry. Apparent KD values were calculated from the dilution series. Figure S2: Dose-response comparison of Privigen and pAbs-Aβ in Thioflavin T assay. Thioflavin T assay showing aggregation kinetics of recombinant Aβ42 (5 µM) alone or co-incubated with either A Privigen at 100 µM, 10 µM and 1 µM or B with pAbs-Aβ at 1 µM, 0.5 µM, 0.1 µM and 0.01 µM. Aβ aggregation was delayed by incubation with Privigen at 100 µM and pAbs-Aβ at 1 µM. Privigen showed no efficacy at 1 µM, pAbs-Aβ lost efficacy at 0.01 µM, indicating that pAbs-Aβ are 100-fold more effective in the Thioflavin T assay than the full IVIG preparation. Figure S3: 6E10 inhibited Aβ-binding to primary cortical neurons but Privigen Fc fragment had no such effect. Staining of rat primary cortical neurons (DIV5) after treatment with 10 µM monomeric Aβ42 and A Privigen Fc fragment at 45 µM or B with 6E10 at 0.5 µM. Confocal images showed that 6E10 at 0.5 µM apparently disrupted the binding of Aβ to the neuronal cell body and dendrites, which resulted in the distribution of Aβ in the medium as immune-complexes. In contrast, incubation with Privigen Fc fragment did not result in the formation of immune-complexes and did not prevent binding of Aβ to the neurons. Microtubule-associated protein 2 (green), Aβ42 (red), antibody test substances (blue), immune-complexes (violet). Bar 100 µm. Figure S4: Blockade of microglial endocytosis of fibrillar Aβ prevents upregulation of CD11b. BV-2 cells were treated for 4 h with 2 µM FITC-labeled Aβ fibrils alone or co-incubated with A Cytochalasin D, B Fucoidan, C Privigen Fc fragment (45 µM) or D Privigen and Cytochalasin D. Incubation with Cytochalasin D, Fucoidan, Privigen Fc fragment and Privigen and Cytochalasin D resulted in markedly reduced uptake of FITC-Aβ fibrils with [file pone.0063162.s001.pdf]
